# Relaxation of thoracic aorta and pulmonary artery rings of marmosets (*Callithrix spp.*) by endothelium-derived 6-nitrodopamine

**DOI:** 10.1590/1414-431X2023e12622

**Published:** 2023-04-07

**Authors:** J. Britto-Júnior, A.T. Lima, J.S. Santos-Xavier, P. Gonzalez, F.Z. Mónica, R. Campos, V.B. de Souza, A.A. Schenka, E. Antunes, G. De Nucci

**Affiliations:** 1 Departamento de Farmacologia, Faculdade de Ciências Médicas, Universidade Estadual de Campinas, Campinas, SP, Brasil; 2 Instituto Superior de Ciências Biomédicas, Universidade Estadual do Ceará, Fortaleza, CE, Brasil; 3 Unidade de Farmacologia Clínica, Centro de Pesquisa e Desenvolvimento de Medicamentos, Universidade Federal do Ceará, Fortaleza, CE, Brasil; 4 Departamento de Farmacologia, Instituto de Ciências Biomédicas, Universidade de São Paulo, São Paulo, SP, Brasil

**Keywords:** Dopamine, Nitric oxide, L-741,626, Electric field stimulation, ODQ

## Abstract

6-Nitrodopamine is a novel catecholamine released by vascular tissues, heart, and vas deferens. The aim of this study was to investigate whether 6-nitrodopamine is released from the thoracic aorta and pulmonary artery rings of marmosets (*Callithrix spp.*) and to evaluate the relaxing and anti-contractile actions of this catecholamine. Release of 6-nitrodopamine, dopamine, noradrenaline, and adrenaline was assessed by liquid chromatography with tandem mass spectrometry (LC-MS/MS). The relaxations induced by 6-nitrodopamine and by the selective dopamine D2 receptor antagonist L-741,626 were evaluated on U-46619 (3 nM)-pre-contracted vessels. The effects of 6-nitrodopamine and L-741,626 on the contractions induced by electric-field stimulation (EFS), dopamine, noradrenaline, and adrenaline were also investigated. Both aorta and pulmonary artery rings exhibited endothelium-dependent release of 6-nitrodopamine, which was significantly reduced by the NO synthesis inhibitor L-NAME. Addition of 6-nitrodopamine or L-741,626 caused concentration-dependent relaxations of both vascular tissues, which were almost abolished by endothelium removal, whereas L-NAME and the soluble guanylate cyclase inhibitor ODQ had no effect on 6-nitrodopamine-induced relaxations. Additionally, pre-incubation with 6-nitrodopamine antagonized the dopamine-induced contractions, without affecting the noradrenaline- and adrenaline-induced contractions. Pre-incubation with L-741,626 antagonized the contractions induced by all catecholamines. The EFS-induced contractions were significantly increased by L-NAME, but unaffected by ODQ. Immunohistochemical assays showed no immunostaining of the neural tissue markers S-100 and calretinin in either vascular tissue. The results indicated that 6-nitrodopamine is the major catecholamine released by marmoset vascular tissues, and it acts as a potent and selective antagonist of dopamine D_2_-like receptors. 6-nitrodopamine release may be the major mechanism by which NO causes vasodilatation.

## Introduction

The nitrocatecholamines 6-nitronoradrenaline and 6-nitroadrenaline have been extracted from rat brain (1,2), and noradrenaline transport in rat synaptosomes is blocked by 6-nitronoradrenaline (1). These nitrocatecholamines have been proposed to be neuronal mediators in the central nervous system, since intrathecal administration of 6-nitronoradrenaline induces analgesia due to release of noradrenaline (3).

6-Nitrodopamine (6-ND) is a novel catecholamine that is released by the endothelium of human cord vessels (4), *Chelonoidis carbonarius* aortic rings (5), and *Pantherophis guttatus* aortic rings (6). In these vascular tissues, 6-ND is a potent vasodilator, acting as a highly selective dopamine D_2_-like receptor antagonist. The synthesis/release of 6-ND is coupled to nitric oxide (NO) synthesis, since it is significantly reduced when the vascular tissues are pre-treated with the NO synthase inhibitor L-NAME. In *Panterophis guttatus* aortic rings, the contractions induced by electric-field stimulation (EFS) are increased by pre-treatment of the rings with either L-NAME or the heme-site soluble guanylate cyclase inhibitor ODQ (7); however, the increased contractions by L-NAME were significantly higher than those caused by ODQ, indicating that synthesis of 6-ND may be the major mechanism responsible for NO-induced vasodilatation (4,5). In addition, 6-ND is an endogenous mediator of human (8) and rat (9) vas deferens contractility, which is blocked by tricyclic antidepressants and by α_1_- and β_1_-adrenoceptor antagonists (10,11). 6-ND is also a potent endogenous modulator of rat heart chronotropism, being 100 times more potent than noradrenaline and adrenaline and 10,000 times more potent than dopamine as a positive chronotropic agent (12).

There is evidence that the non-human primate and New World monkey *Callithrix jacchus* (marmoset) can be rather close to the human situation at the structural and molecular level (13). Thus, we investigated whether 6-ND is released from the thoracic aorta and pulmonary artery rings of marmosets (*Callithrix spp.*) and what effect it has on these vascular tissues.

## Material and Methods

### Animals

All experimental procedures on marmosets (*Callithrix spp.*) of both sexes were approved by the Institutional Animal Care and Use Committee (CEUA/UNICAMP: 5203-1/2019) and followed the Brazilian Guidelines for the Production, Maintenance and Use of Animals for Teaching or Research from the National Council of Control in Animal Experimentation (CONCEA), and the ARRIVE guidelines. The use of marmosets (*Callithrix spp.*) was authorized by the Brazilian Institute for Environment (Sisbio number 75201-3), and the animals were provided by the Parque Ecológico do Tietê (Brazil).

### Basal release of 6-ND, dopamine, noradrenaline, and adrenaline

Adult marmosets (260-310 g) were anesthetized with ketamine and xylazine (80 mg/kg *im* and 10 mg/kg *im*, respectively) after sedation with midazolam (2 mg/kg *im*). Exsanguination was performed to confirm the euthanasia. After euthanasia, the thoracic aorta and pulmonary artery were removed, with special care not to damage the endothelial layer or to over distend the vessels during the procedure, and placed in containers with Krebs Henseleit solution (KHS, in mM: NaCl 118, KCl 4.7, CaCl_2_ 2.5, MgSO_4_ 1.2, NaHCO_3_ 25, KH_2_PO_4_ 1.2, and dextrose 5.6; pH 7.4, 37°C, 95% O_2_/5% CO_2_). To investigate the release of catecholamines from the thoracic aorta, one entire thoracic aorta was placed in 5-mL organ baths containing KHS solution with ascorbic acid (1 mM) at 37°C for 30 min. When required, the removal of the thoracic aorta endothelium was performed by gently rubbing the vessels with forceps. The endothelium-intact isolated aortic rings were incubated in the absence and presence of the NO synthesis inhibitor N^w^-nitro-L-arginine (L-NAME, 100 μM). A 2-mL KHS aliquot was transferred to a tube and stored at -20°C until further analysis by liquid chromatography with tandem mass spectrometry (LC-MS/MS; 5). To investigate the release of catecholamines from the pulmonary artery, two pulmonary arteries (obtained from two animals) were placed in 5-mL organ baths containing KHS with ascorbic acid (1 mM) at 37°C for 30 min, and the same procedures described above for the thoracic aorta were followed.

### Determination of catecholamine concentrations

The method employed for 6-ND quantification (14) was modified to allow the measurement of the four catecholamines in a single chromatographic run. Briefly, the extraction of the catecholamines from KHS (1 mL) was performed by solid phase extraction. To 1 mL of KHS was added 50 mL (100 ng/mL) of the deuterated catecholamines used as internal standards, and the samples were homogenized for 10 s. The Strata™-X 33 mm Polymeric Reversed Solid Phase Extraction (SPE) cartridges (Phenomenex Inc, USA) were pre-washed with MeOH (1 mL) followed by deionized H_2_O (2 mL). After sample introduction into the cartridge, the cartridge was subsequently washed 3 times with deionized H_2_O. The catecholamines were then eluted with 900 mL MeOH/H_2_O (90/10, v/v) with formic acid (0.1%). The eluate was evaporated under N_2_ flow at 50°C. The residue was dissolved with 100 mL of acetonitrile/H_2_O (50/50, v/v) with 0.1% formic acid and transferred to vials ready for injection into the mobile phase (75% A solution, composed of deionized H_2_O with 0.1% formic acid (v/v) and 25% B solution, composed of acetonitrile/H_2_O (90/10, v/v) with 0.1% formic acid. The mobile phase perfused a LC ADVp Liquid Chromatograph Shimadzu System (Shimadzu, Japan) coupled to a Shimadzu 8060 triple quadrupole mass spectrometer operating in ESP^+^ mode at 350 mL/min. The dissolved residues were injected by a SIL-30AC autoinjector (Shimadzu), at a temperature of 8°C. The transitions monitored by electrospray multiple reaction monitoring (MRM), injection volume, run-time, and limit of quantitation were described elsewhere (9). The results are reported as means±SE.

### Preparations for isometric tension recordings

The thoracic aorta and pulmonary artery rings (3-mm length) were suspended vertically between two metal hooks in 10-mL organ baths containing KHS, gassed with a mixture of 95% O_2_ and 5% CO_2_ (pH 7.4) at 37°C. Isometric force was recorded using a PowerLab 400TM data acquisition system (Software Chart, version 7.0, AD Instrument, USA). The tissues were allowed to equilibrate for 1 h before starting the protocols, as detailed below.

### Aorta and pulmonary artery relaxation responses to 6-ND and L-741,626

Endothelium-intact and endothelium-denuded aortic and pulmonary artery rings were pre-contracted with the thromboxane A_2_ (TXA_2_) mimetic U-46619 (3 nM). The integrity of the endothelium in both vessels was evaluated through ATP-induced relaxation (ATP, 10 µM). In endothelium-intact rings, after a sustained contraction was obtained, cumulative concentration-response curves to either 6-ND (10 pM-1 µM) or the selective dopamine D_2_-receptor antagonist L-741,626 (10 pM^-1^ µM) were performed in the presence or absence of L-NAME (100 µM) or ODQ (100 μM). Concentration-response curves to either 6-ND (10 pM-1 µM) and L-741,626 (10 pM-1 µM) were also performed in endothelium-denuded rings.

### Aorta and pulmonary artery contractions to EFS

In separate experiments, endothelium-intact thoracic aorta and pulmonary rings were submitted to EFS at 60 V for 30 s, at 8-32 Hz in square-wave pulses, 0.3 ms pulse width, 0.1 ms delay, using a Grass S88 stimulator (Astro-Medical, USA). The EFS-induced contractions of the thoracic aorta and pulmonary rings were performed in preparations pre-treated (30 min) with L-NAME (100 µM) or ODQ (100 μM). The EFS-induced contractions were also evaluated in the presence of 6-ND (1 μM) or L-741,626 (1 μM). Potassium chloride (KCl, 80 mM) was added at the beginning and at the end of the experimental protocols to evaluate tissue reactivity after EFS (5).

### Aorta and pulmonary artery contractions to dopamine, noradrenaline, and adrenaline

In endothelium-intact thoracic aortic rings pre-treated with L-NAME (100 µM; 30 min), cumulative concentration-response curves to dopamine (1 nM-300 μM), noradrenaline (1 nM-100 µM), and adrenaline (1 nM-100 µM) were carried out in the absence and in the presence of either 6-ND (0.1, 0.3, and 1 μM) or L-741,626 (0.1, 0.3, and 1 μM).

### Immunohistochemistry for S-100 and calretinin

Following euthanasia, pulmonary artery and aorta (n=3 for each vessel) were collected, fixed in 10% neutral buffered formalin for 24 h at 24°C, dehydrated, embedded in paraffin wax, and sectioned in 4-μm sections. Subsequently, these sections were immunostained for S-100 protein (a neural tissue marker) or calretinin (a neural/neuronal marker) to investigate the presence of nerve fibers within vascular walls using the following primary antibodies: anti-S-100 (mouse monoclonal antibody, ab4066, at 1:100, Abcam, USA) and anti-calretinin (rabbit monoclonal antibody; ab92341; at 1:100, Abcam).

Immunohistochemistry was performed manually. Briefly, the sections were deparaffinized in xylene and rehydrated in a series of ethanol baths of increasing concentrations. They were then incubated in citrate buffer at pH 6.0 in a steamer set for 20 min (at 95°C). The sections were then incubated for 2 h at room temperature (25°C) with the above-mentioned primary antibodies. Subsequently, these sections were incubated with the NovoLink Max Polymer Detection System (Novocastra/Leica Biosystems, USA), following the manufacturer's instructions, and using diaminobenzidine (liquid DAB, DakoCytomation, USA) as a chromogen (which renders a brown precipitate at the antibody binding site). Finally, the sections were counter-stained with Harris' hematoxylin and cover-slipped in Entellan medium (Merck, Germany).

Negative controls consisted of omission of the primary antibody and incubation with the primary antibody diluents and with the detection system. This was performed for all the immunohistochemical assays to identify any background staining. Formalin-fixed, paraffin-embedded marmoset brain sections (n=3) and human cerebellum sections (n=1) were used as positive controls for the presence of both antigens (i.e., S-100 protein and calretinin). All slides were examined and photomicrographed using a trinocular Eclipse 50i microscope (Nikon, Japan) coupled to a 10MP CMOS digital camera (AmScope, USA). Positivity was assessed by an experienced MD, PhD pathologist (A.A.S.), who was blind to the presence/absence of the primary antibody on the sample under examination (the observer did not know whether a test sample or an omission control was being assessed). Blinding was achieved by covering the slide labels with a removable occluding sticker.

### Data analysis

Nonlinear regression analysis was carried out to determine the half maximal effective concentration (pEC_50_) using GraphPad Prism (GraphPad Software, version 9.4, USA) with the constraint that F=0. All concentration-response data were evaluated for a fit to a logistics function in the form: E = E_max_ / ([1 + (10c / 10x) n] + F, where E is the increase in contractile response induced by the agonist, E_max_ is the effect agonist maximum, c is the logarithm of concentration of the agonist that produces 50% of E_max_, x is the logarithm of the concentration of the drug; the exponential term n is a curve-fitting parameter that defines the slope of the concentration-response line, and F is the response observed in the absence of added drug. The EC_50_ data are reported as means±SE of n experiments. Values of E_max_ are reported in mN (contractile protocols) or percent levels of relaxations of the U-46619-induced pre-contraction (relaxation protocols). One ring was used as the response control and the other ring was incubated with an antagonist/inhibitor. Data are reported as means±SE of the number of experiments. In the pharmacological experiments, the number of experiments (n) is reported as x/y, where x is the number of animals and y, the number of rings employed. The contractions were quantified in milli-Newtons (mN) whereas the relaxant responses are reported as a percentage of the level of pre-contraction achieved with U-46619. For E_max_ and pEC_50_ analysis, two-tail unpaired Student's *t*-test was used and the difference between groups at P<0.05 was considered significant. The pA_2_ values of the antagonists were calculated by the equation: pA_2_ = log (antagonist concentration) − log (CR-1) − log (antagonist concentration) (15) (CR = concentration ratio).

### Drugs and solutions

Dopamine, N^ω^-nitro-L-arginine methyl ester hydrochloride (L-NAME), and ascorbic acid were obtained from Sigma-Aldrich Chemicals Co. (USA). Adrenaline, L-741,626, noradrenaline, ^1^H-(1,2,4)oxadiazolo[4,3-a]quinoxalin-1-one (ODQ), and U-46619 were purchased from Cayman Chemical Co. (USA). 6-Nitrodopamine and 6-nitrodopamine-d_4_ were acquired from Toronto Research Chemicals (Canada). Dopamine‐d_3_ hydrochloride, DL‐noradrenaline‐d_6_ hydrochloride, and adrenaline‐d_6_ hydrochloride were acquired from CDN Isotopes (Canada). Strata™-X 33 mm Polymeric Reversed SPE cartridges were bought from Phenomenex (USA) and GIST-HP C_18_ columns were obtained from Shimadzu (Germany). Sodium chloride (NaCl), potassium chloride (KCl), calcium chloride (CaCl_2_), magnesium sulfate (MgSO_4_), sodium bicarbonate (NaHCO_3_), potassium phosphate monobasic (KH_2_PO_4_), and glucose were acquired from Merck KGaA (Germany). Anti-S-100 (mouse monoclonal antibody, ab4066) and anti-calretinin (rabbit monoclonal antibody; ab92341) were obtained from Abcam.

## Results

### Catecholamine release

**Figure 1:**
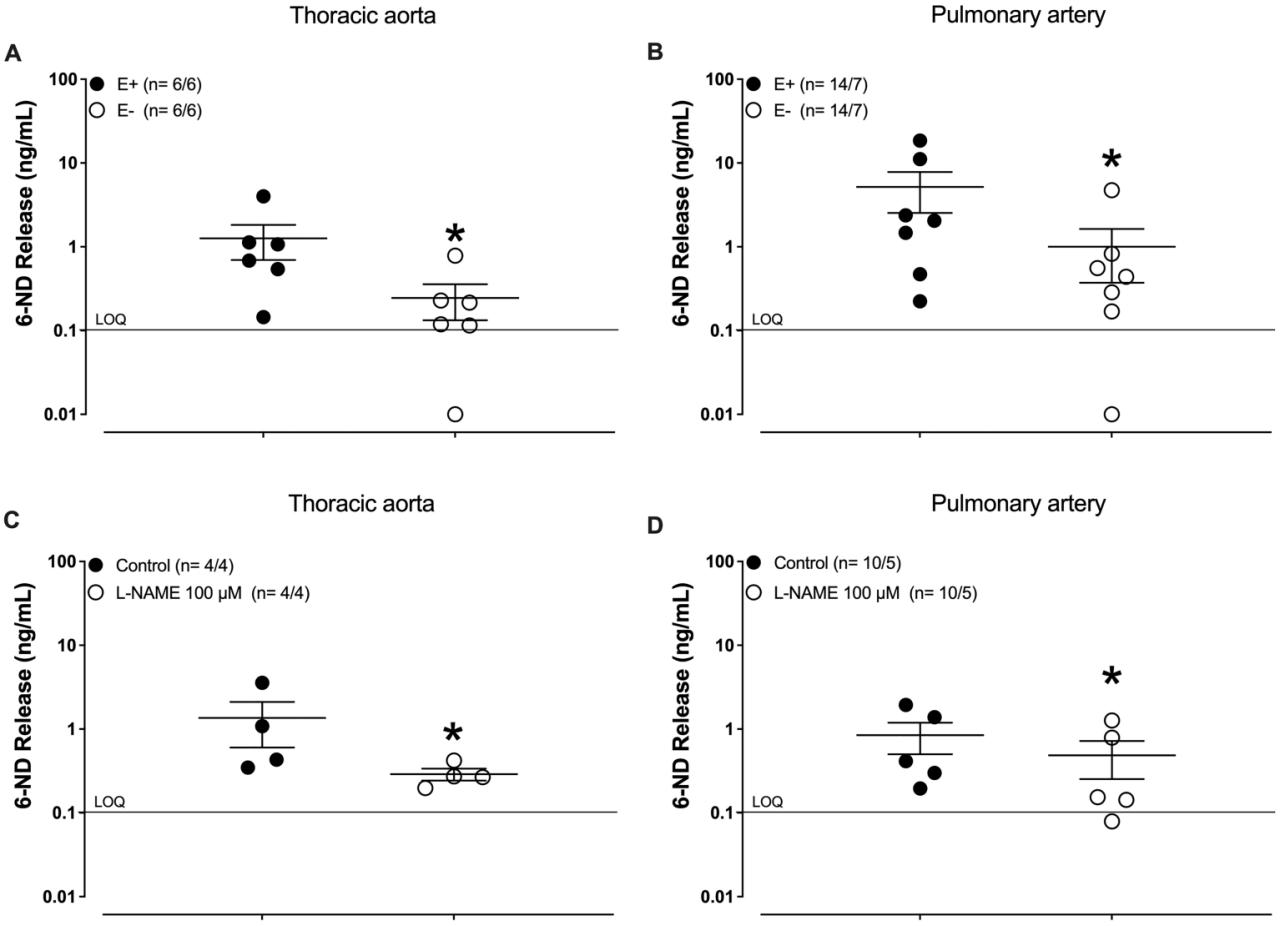
Basal release of 6-nitrodopamine (6-ND) from thoracic aorta and pulmonary artery rings. Panels **A** and **B** show the effect of endothelium removal (E-) on the basal release of 6-ND from thoracic aorta (n=6/6) and pulmonary artery (n=14/7), respectively. Panels **C** and **D** show the effect of pre-incubation (30 min) of L-NAME (100 µM) on basal release of 6-ND from thoracic aorta (n=4/4) and pulmonary artery (n=14/7), respectively. The number of experiments (n) is reported as x/y, where x represents the number of animals and y the number of rings employed. Data are reported as means±SE. *P<0.05 (Student's unpaired *t*-test).

Marmoset thoracic aorta (Figure 1A and C) and pulmonary artery rings (Figure 1B and D) presented basal release of 6-ND, as detected by LC-MS/MS. The basal release of 6-ND was significantly reduced in endothelium-denuded thoracic aorta (Figure 1A), endothelium-denuded pulmonary artery (Figure 1B), and in the vascular tissues pre-treated (30 min) with L-NAME (100 µM; Figure 1C and D).

In endothelium-intact thoracic aorta rings, the release of dopamine, noradrenaline, and adrenaline were below the limit of quantitation (LOQ, data not shown). In endothelium-intact pulmonary artery rings, basal release of dopamine was observed in all samples (3.5±1.6 ng/mL, n=7) and was significantly reduced in endothelium-denuded pulmonary artery rings (0.8±0.6 ng/mL, n=7). In endothelium-intact pulmonary artery rings, basal release of noradrenaline was observed only in 3 out of 7 experiments (0.4±0.2 ng/mL), and basal release of adrenaline was below the LOQ in all samples.

### Relaxant effect of 6-ND and L-741,626 on pre-contracted rings

**Figure 2:**
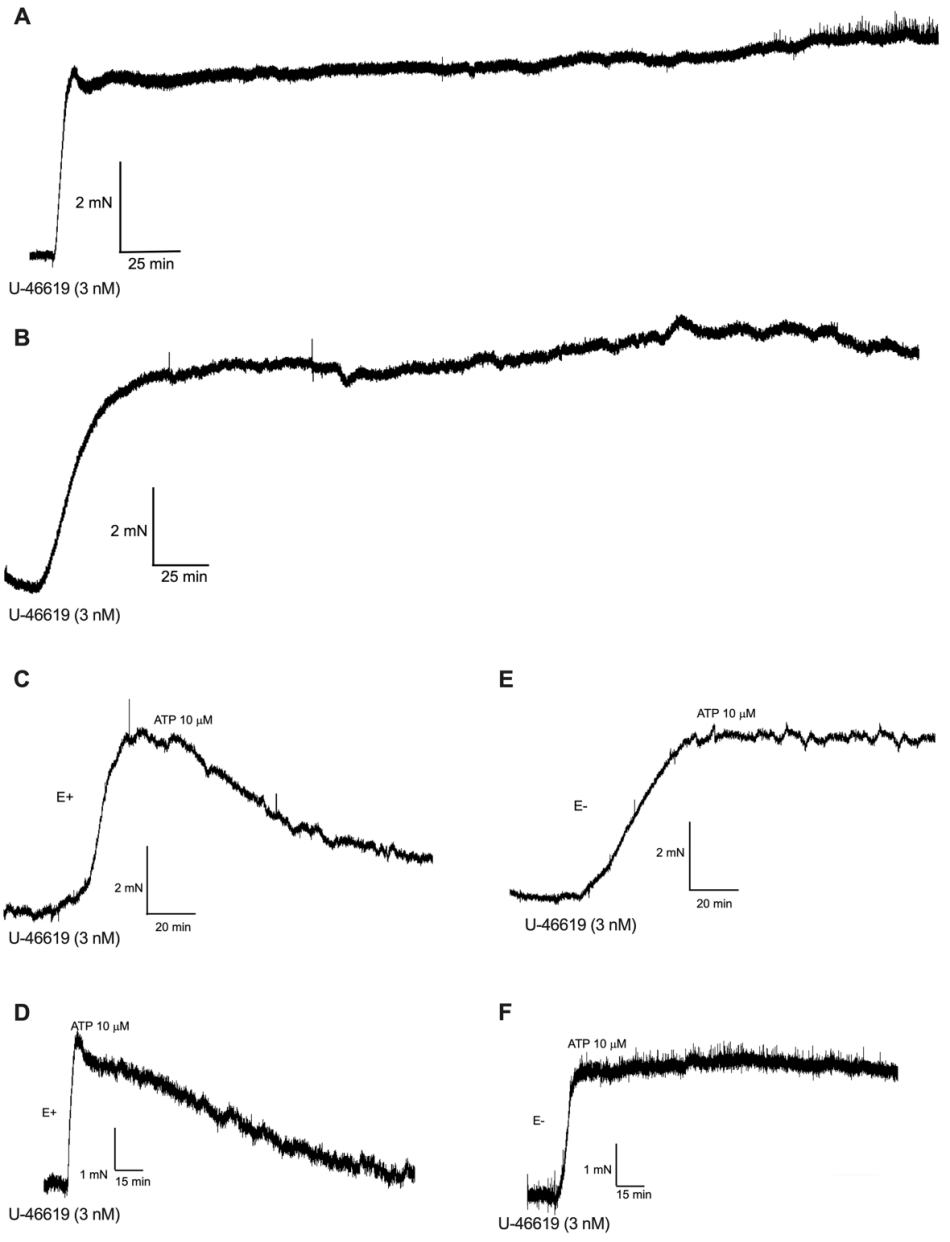
Contractions induced by U-46619 of marmoset arteries. The endoperoxide analogue U-46619 (3 nM) induced a stable and durable contraction of both thoracic aorta (**A**) and pulmonary artery (**B**) rings. In endothelium-intact thoracic aorta (**C**) and pulmonary artery (**D**) rings, pre-contracted with U-46619 (3 nM), adenosine triphosphate (ATP; 10 µM) caused relaxations that were not observed when the endothelium was mechanically removed from the thoracic aorta (**E**) and pulmonary artery (**F**) rings.

The endoperoxide analogue U-46619 (3 nM) induced a stable and lasting contraction of both thoracic aorta (Figure 2A) and pulmonary artery (Figure 2B) rings. ATP (10 µM) caused relaxation of both pre-contracted thoracic aorta (Figure 2C) and pulmonary artery (Figure 2D) rings, which were inhibited when the endothelium was removed from thoracic aorta (Figure 2E) and pulmonary artery (Figure 2F) rings.

**Figure 3:**
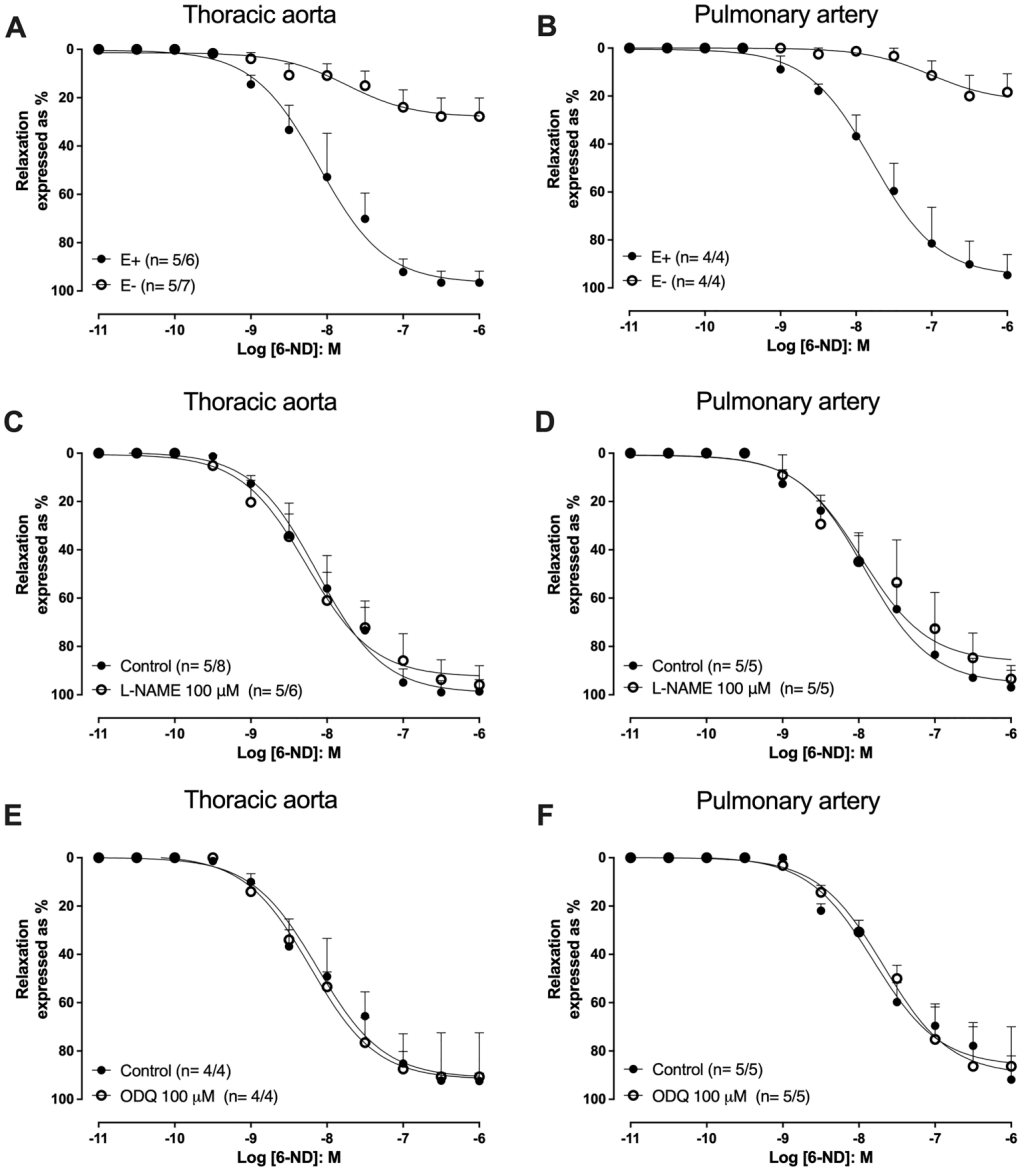
Relaxations induced by 6-nitrodopamine (6-ND) in U-46619-pre-contracted thoracic aorta and pulmonary artery rings. Panels **A** and **B** show the effect of endothelium removal (E-) on the relaxations induced by 6-ND on thoracic aorta (n=5/7) and pulmonary artery rings (n=4/4), respectively. Panels **C** and **D** show the effect of pre-incubation (30 min) of L-NAME (100 µM) on the relaxations induced by 6-ND on thoracic aorta rings (n=5/6) and pulmonary artery rings (n=5/5), respectively. Panels **E** and **F** show the effect of pre-incubation (30 min) of ODQ (100 μM) on the relaxations induced by 6-ND on thoracic aorta rings (n=4/4) and pulmonary artery rings (n=5/5), respectively. The number of experiments (n) is reported as x/y, where x represents the number of animals and y the number of rings employed.

In U-46619 (3 nM) pre-contracted rings with intact endothelium, 6-ND (10 pM-1 µM) induced concentration-dependent relaxations in the thoracic aorta (Figure 3A, C, and E; pEC_50_ of 8.10±0.12) and pulmonary artery rings (Figure 3B, D, and F; pEC_50_ of 7.78±0.06). In endothelium-denuded rings, the relaxations induced by 6-ND were markedly reduced in both thoracic aorta (Figure 3A) and pulmonary artery rings (Figure 3B). In preparations with intact endothelium, the 6-ND-induced relaxations were affected neither by pre-treatment (30 min) with L-NAME (100 µM; Figure 3C and D) nor by ODQ (100 µM; Figure 3E and F).

**Figure 4:**
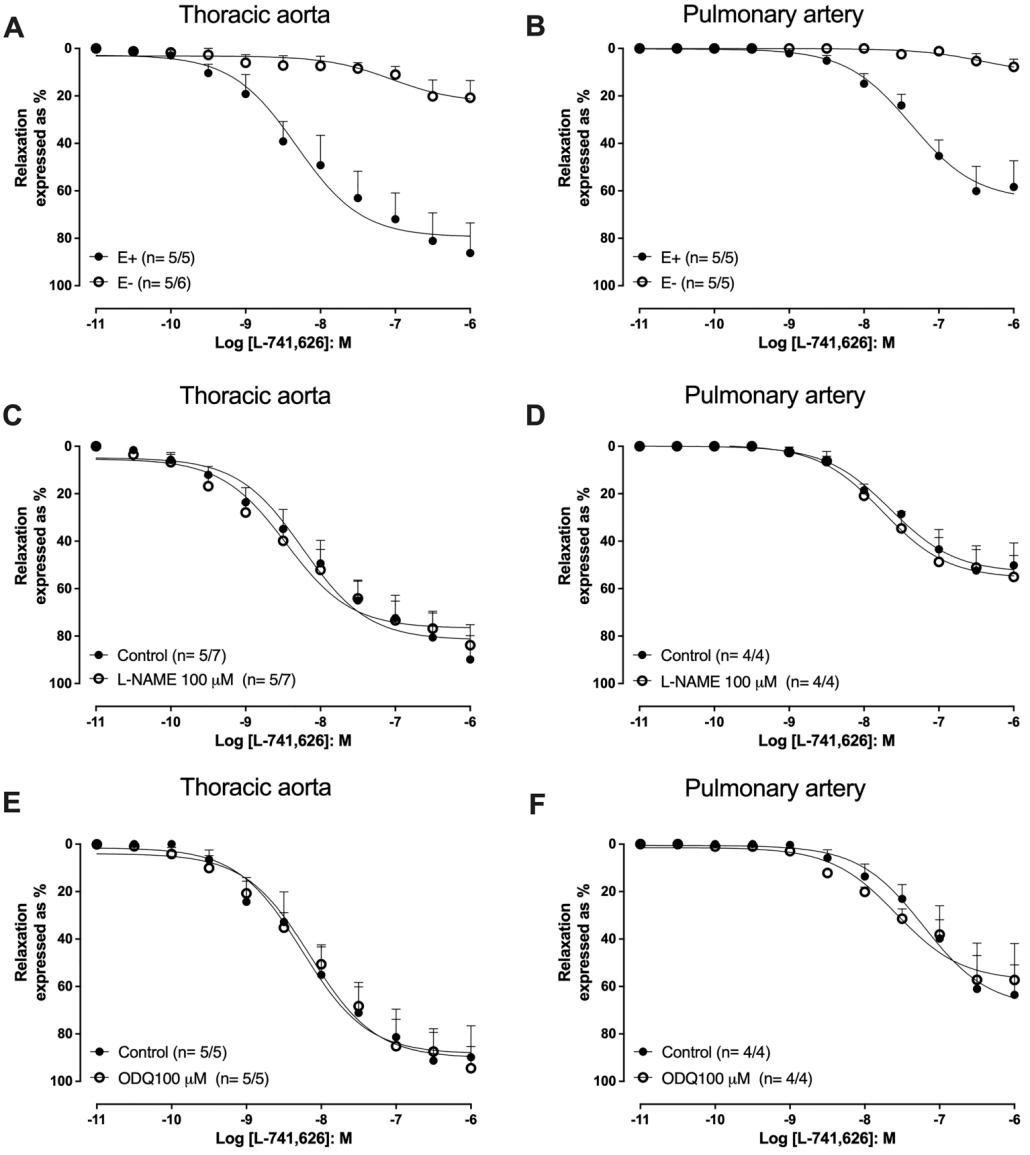
Relaxations induced by L-741,626 in U-46619 pre-contracted thoracic aorta and pulmonary artery rings. Panels **A** and **B** illustrate the effect of endothelium removal (E-) on the relaxations induced by L-742,626 on thoracic aorta (n=5/5) and pulmonary artery rings (n=5/5), respectively. Panels **C** and **D** show the effect of pre-incubation (30 min) of L-NAME (100 μM) on the relaxations induced by selective dopamine D_2_-receptor antagonist L-742,626 on thoracic aorta rings (n=5/7) and pulmonary artery rings (n=4/4), respectively. Panels **E** and **F** show the effect of pre-incubation (30 min) of ODQ (100 μM) on the relaxations induced by L-742,626 on thoracic aorta rings (n=5/5) and pulmonary artery rings (n=4/4), respectively. The number of experiments (n) is reported as x/y, where x represents the number of animals and y the number of rings employed.

The dopamine D_2_ receptor antagonist L-741,626 (10 pM-1 µM) induced concentration-dependent relaxations in the thoracic aorta (Figure 4C, and E; pEC_50_ of 8.32±0.17) and pulmonary artery rings (Figure 4B, D, and F; pEC_50_ of 7.37±0.07). In endothelium-denuded rings, the relaxations induced by L-741,626 were almost abolished in both the thoracic aorta (Figure 4A) and pulmonary artery rings (Figure 4B). The L-741,626-induced relaxations were affected neither by pre-treatment (30 min) with L-NAME (100 µM; Figure 4C and D) nor with ODQ (100 µM; Figure 4E and F).

### Effect of 6-ND, L-741,626, L-NAME, and ODQ on EFS-induced aortic and pulmonary artery contractions

**Figure 5:**
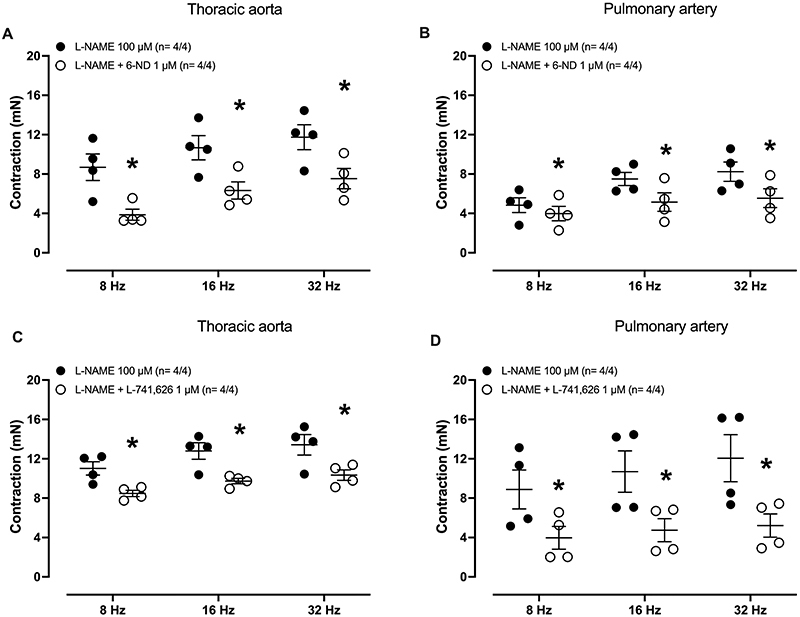
Effect of 6-nitrodopamine (6-ND) and L-741,626 on electric-field stimulation (EFS)-induced contractions in thoracic aorta and pulmonary artery rings. Endothelium-intact aorta and pulmonary artery rings were pre-treated with L-NAME (100 µM, 30 min), after which were incubated or not with either 6-ND (1 μM, n= 4/4; Panels **A** and **B**) or the selective dopamine D_2_-receptor antagonist L-741,626 (1 µM, n= 4/4; Panels **C** and **D**). EFS at 8 to 32 Hz was then applied to both tissues. The number of experiments (n) is reported as x/y, where x represents the number of animals and y the number of rings employed. Data are reported as means±SE. *P<0.05 (Student's unpaired *t*-test).

**Figure 6:**
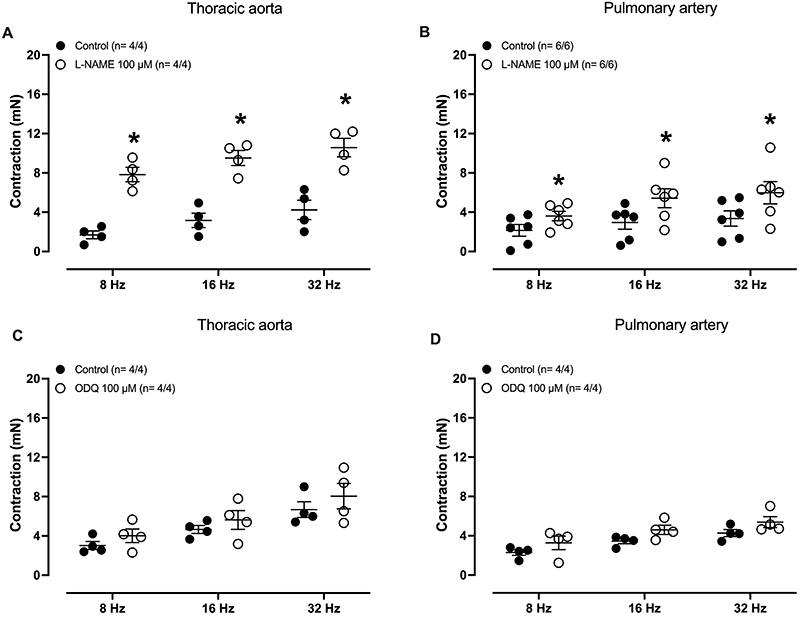
Effect of L-NAME and ODQ on electric-field stimulation (EFS)-induced thoracic aorta and pulmonary artery contractions. Panels **A** and **B** show the effect of pre-incubation (30 min) of L-NAME (100 μM) on the contractions induced by EFS (8, 16, and 32 Hz) on thoracic aorta rings (n=4/4) and pulmonary artery rings (n=6/6), respectively. Panels **C** and **D** show the effect of pre-incubation (30 min) of ODQ (100 μM) on the contractions induced by EFS on thoracic aorta rings (n=4/4) and pulmonary artery rings (n=4/4), respectively. The number of experiments (n) in each panel is reported as x/y, where x represents the number of animals and y the number of rings employed. Data are reported as means±SE. *P<0.05 (Student's unpaired *t*-test).

In endothelium-intact thoracic aorta (Figure 5A and C) and pulmonary artery rings (Figure 5B and D) pre-treated (30 min) with L-NAME (100 µM), applying EFS caused frequency-dependent (8-32 Hz) contractions (Figure 5A-D), which were significantly reduced when the tissues were previously (30 min) incubated with either 6-ND (1 μM, Figure 5A and B) or L-741,626 (1 µM, Figure 5C and D). In addition, in these preparations, EFS-induced aortic and pulmonary artery contractions were significantly increased by pre-treatment (30 min) with L-NAME (100 μM, 30 min; Figure 6A and B), whereas ODQ (100 μM) had no significant effect (Figure 6C and D).

### Effect of 6-ND and L-741,626 on dopamine-, noradrenaline-, and adrenaline-induced thoracic aorta ring contractions

**Figure 7:**
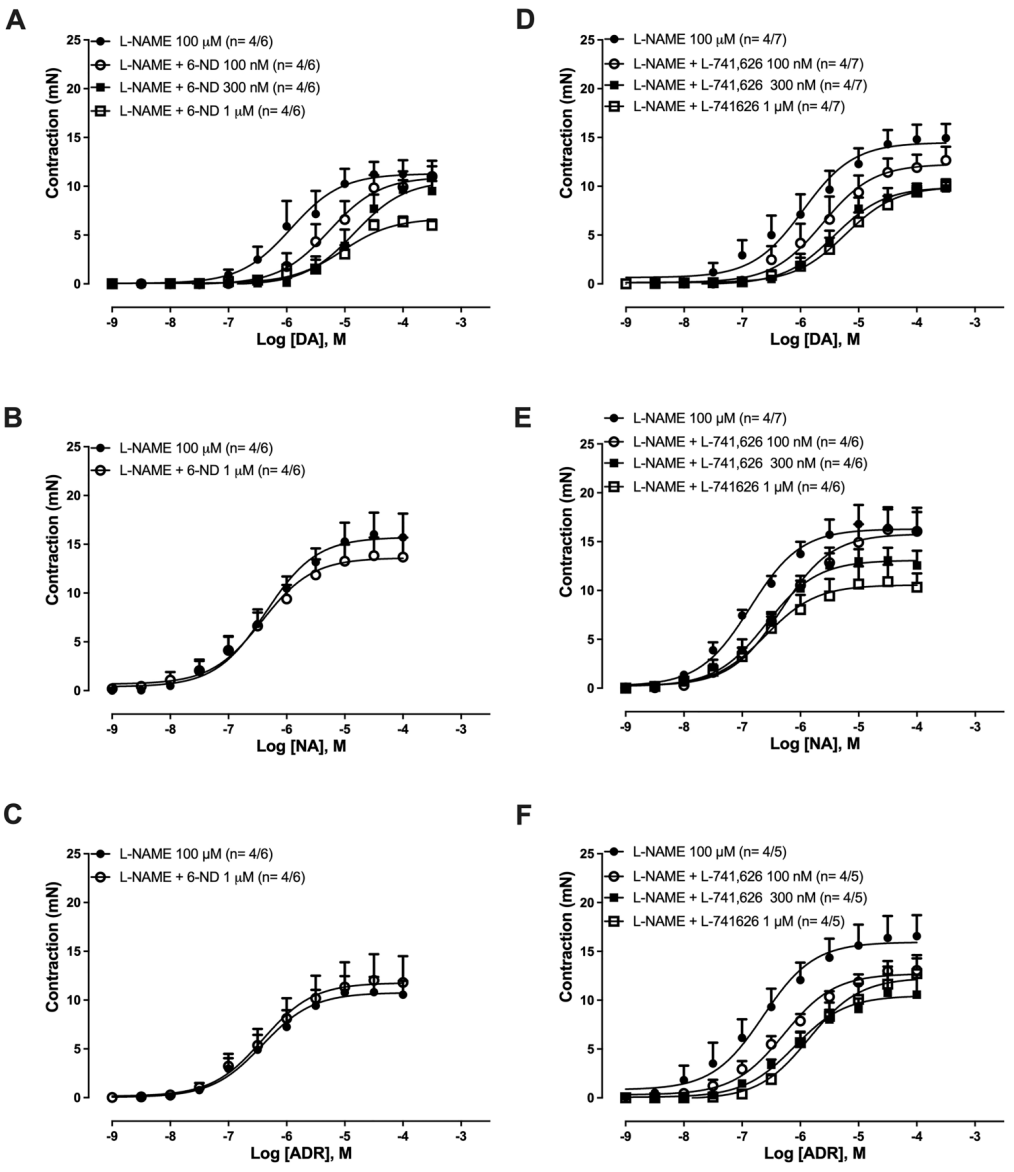
Effect of 6-nitrodopamine (6-ND) and the selective dopamine D_2_-receptor antagonist L-741,626 on dopamine-, noradrenaline-, and adrenaline-induced aortic contractions. Endothelium-intact aortic rings were all pre-treated with L-NAME (100 µM, 30 min), after which were incubated or not with either 6-ND (Panels **A**, **B**, and **C**) or L-741,626 (Panels **D**, **E**, and **F**). Concentration-response curves to dopamine (DA), noradrenaline (NA), and adrenaline (ADR) were then performed. The number of experiments (n) in each panel is reported as x/y, where x represents the number of animals and y the number of rings employed. Data are reported as means±SE.

In endothelium-intact thoracic aorta rings pre-treated with L-NAME (100 μM), addition of dopamine (Figure 7A and D), noradrenaline (Figure 7B and E), or adrenaline (Figure 7C and F) induced concentration-dependent contractions. In these preparations, addition of 6-ND (0.1-1 μM) caused concentration-dependent rightward shifts of the dopamine-induced contractions (Figure 7A; pA_2_ 7.64±0.15), whereas the contractions induced by noradrenaline (Figure 7B) and adrenaline (Figure 7C) remained unaffected by 6-ND (1 μM).

In endothelium-intact thoracic aorta rings pre-treated with L-NAME (100 µM), pre-incubation (30 min) with the dopamine D_2_ receptor antagonist L-741,626 (100 nM-1 µM) caused concentration-dependent rightward shifts of the contractions induced by dopamine (Figure 7D; pA_2_ 7.70±0.15). In contrast to 6-ND, pre-incubation (30 min) with L-741,626 (100 nM-1 μM) caused significant concentration-dependent rightward shifts of the contractions induced by noradrenaline (Figure 7E; pA_2_ 6.94±0.23) and adrenaline (Figure 7F; pA_2_ 6.89±0.14).

### Immunohistochemistry

**Figure 8:**
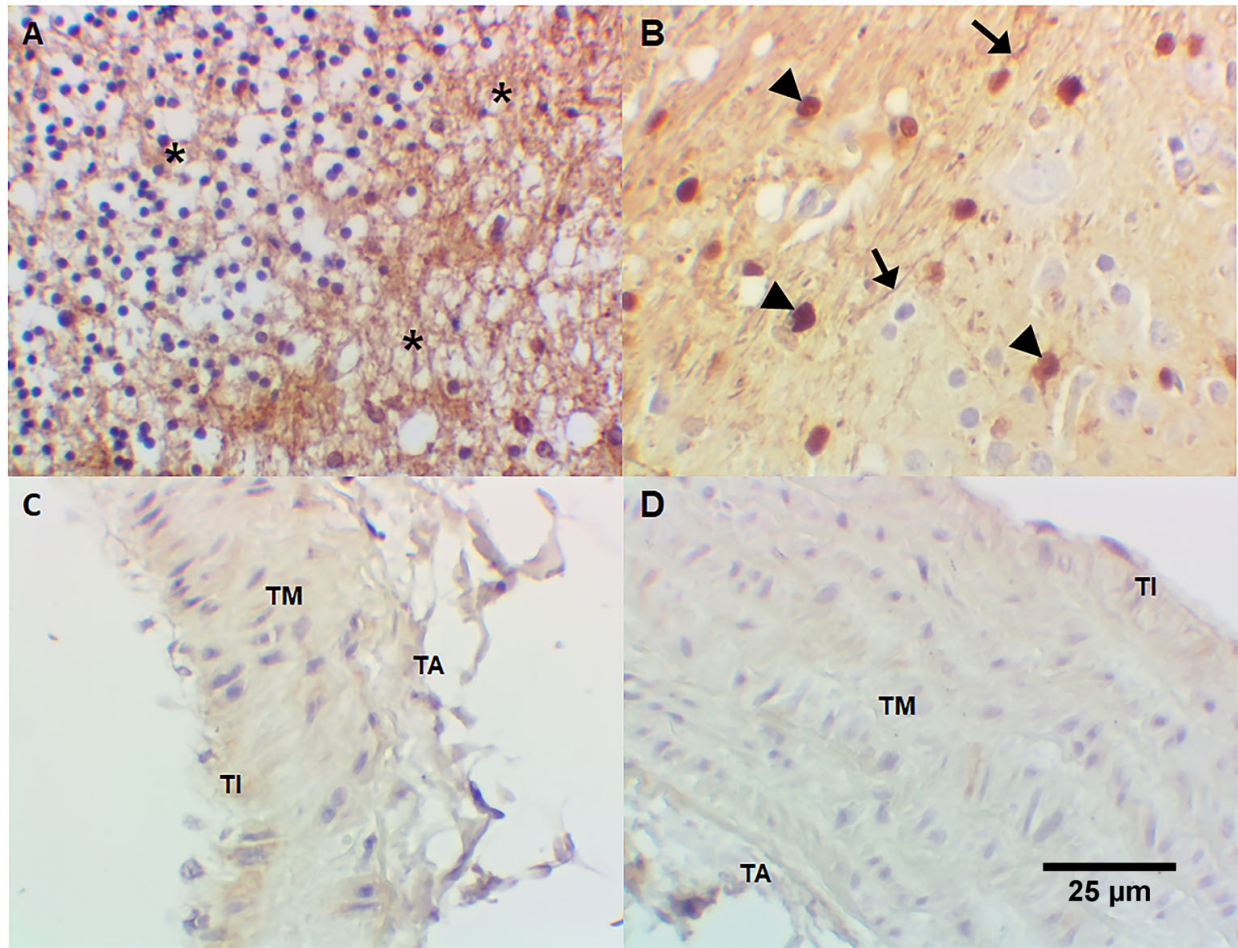
Detection of S-100 protein (neural/neuronal markers) by immunohistochemistry: **A**, diffuse positivity for S-100 protein in human cerebellum neuropil (*); **B**, S-100 protein positivity in *Callithrix spp.* central nervous system (glial nuclei [arrowhead] and glial fibers [black arrow] are positive); **C**, absence of S-100 protein in tunica intima (TI), media (TM), and adventitia (TA) of *Callithrix spp.* pulmonary artery; **D**, absence of S-100 protein in *Callithrix spp.* aorta (both tunicae intima and media are negative). Immunoperoxidase, 400× (original magnification), scale bar 25 µm.

**Figure 9:**
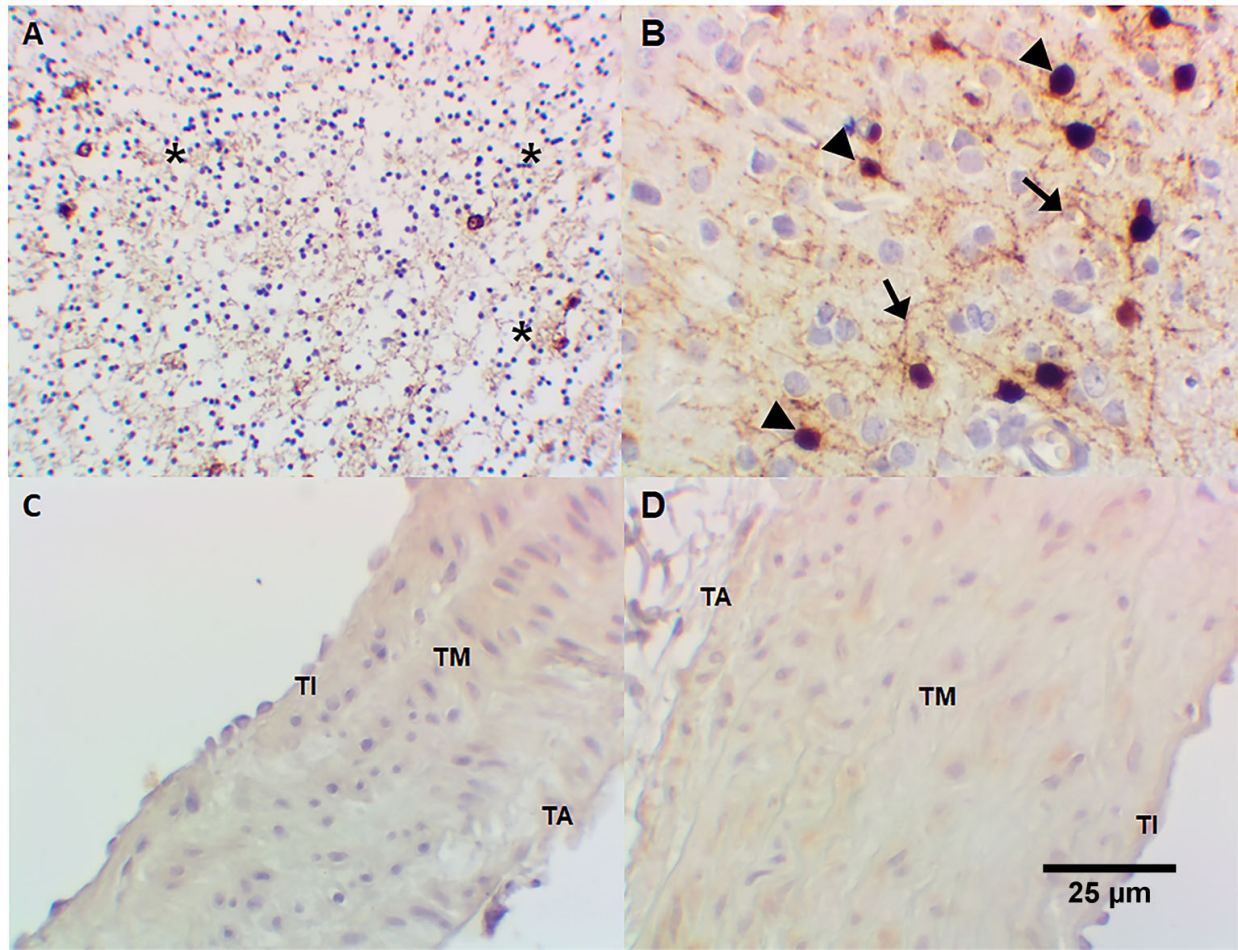
Detection of calretinin (neural/neuronal markers) by immunohistochemistry: **A**, diffuse positivity for calretinin in human cerebellum neuropil (*); **B**, calretinin positivity in *Callithrix spp.* central nervous system (glial nuclei [arrowhead] and glial fibers [black arrow] are positive); **C**, absence of calretinin in tunica intima (TI), media (TM), and adventitia (TA) of *Callithrix spp.* pulmonary artery; **D**, absence of calretinin in *Callithrix spp.* aorta (both tunicae intima and media are negative). Immunoperoxidase, 400× (original magnification), scale bar 25 µm.

**Figure 10:**
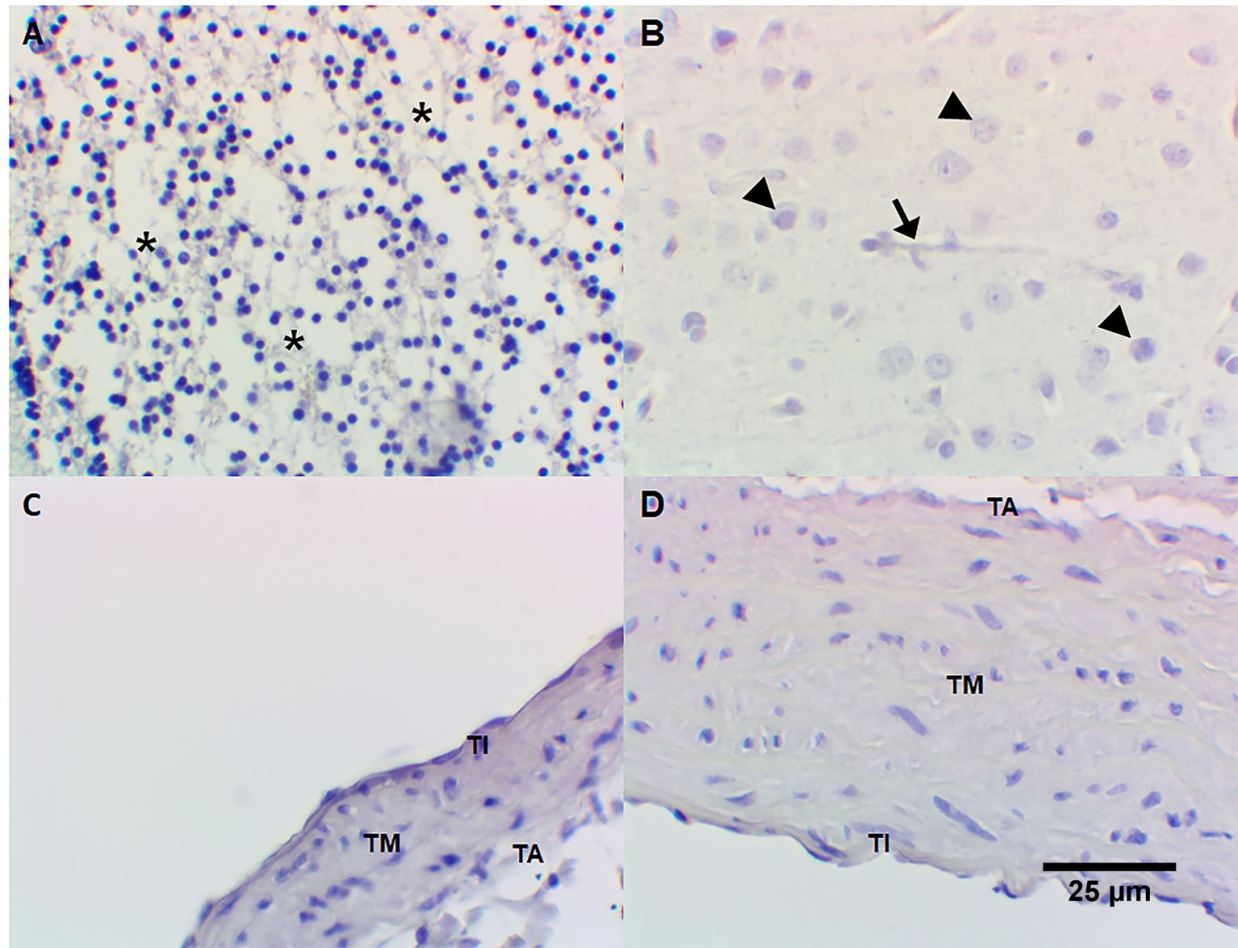
Negative controls (omission of anti-S-100 and anti-calretinin). **A**, Human cerebellum; **B**, *Callithrix spp.* brain; **C**, pulmonary artery of *Callithrix*; **D**, aorta of *Callithrix*. TI: tunica intima; TM: tunica media; TA: tunica adventitia. These images show absence of immunostaining in neuropil (*), glial nuclei (arrowhead), and neuron axons (black arrow). Immunoperoxidase, 400× (original magnification), scale bar 25 µm.

Immunoreactivity for S-100 protein (neural/neuronal neuromarker) was detected in human cerebellum neuropil (Figure 8A) and in *Callithrix spp.* central nervous system (Figure 8B). No immunoreactivity for S-100 protein was observed in tunica intima, tunica media, and adventitia or *Callithrix spp.* pulmonary artery (Figure 8C) and aorta tunica intima and tunica media (Figure 8D). Immunoreactivity for calretinin, another neural/neuronal marker, was positive in human cerebellum neuropil (Figure 9A) and *Callithrix spp.* central nervous system (Figure 9B). No immunoreactivity for calretinin was observed in tunica intima, tunica media, and adventitia or *Callithrix spp.* pulmonary artery (Figure 9C) and the aorta tunica intima and tunica media (Figure 9D). Omission of anti-S-100 protein antibody and anti-calretinin antibody (negative controls) revealed no immunostaining in human cerebellum (Figure 10A), *Callithrix spp.* brain (Figure 10B), *Callithrix spp.* pulmonary artery (Figure 10C), and in *Callithrix spp.* aorta (Figure 10D). The results are summarized in Table 1.

**Table 1:** Immunohistochemical detection of S-100 protein and calretinin in *Callithrix spp.* (marmoset) brain, pulmonary artery, and thoracic aorta.

Sample	Negative control(primary Ab omission)	Antibody (Ab)	
		S-100	Calretinin
Dilution	Omitted	1:100	1:100
Human cerebellum (positive control tissue), n=1	Negative	(+)	(+)
*Callithrix spp.* Brain (species-specific positive control tissue), n=3	Negative	(+)	(+)
*Callithrix spp.* pulmonary artery (target/test tissue), n=3	Negative	(-) in tunicae intima and media	(-) in tunicae intima and media
*Callithrix spp.* Aorta (target/test tissue), n=3	Negative	(-) in tunicae intima and media	(-) in tunicae intima and media

## Discussion

The results clearly showed that 6-ND is the major catecholamine released from marmoset thoracic aortic and pulmonary artery rings. The basal release of 6-ND was significantly reduced in the presence of L-NAME, indicating that NO synthesis played a major role in the biosynthetic pathway of 6-ND. Pre-incubation of the tissues with L-NAME does not abolish 6-ND release in human umbilical cord vessels (4), in *Chelonoidis carbonaria* aortic rings (5) and in *Panterophis guttatus* aortic rings (6). Similar results were observed in rat isolated atrium (12), rat vas deferens (9), and human vas deferens (8). In the case of both rat vas deferens and rat isolated atrium, even chronic treatment with L-NAME did not abolish the basal release of 6-ND. Acute intra-peritoneal administration of L-NAME only attenuated the amounts of 6-nitronoradrenaline extracted from rat brain (1). Whether 6-ND biosynthesis is the result of direct nitrosation of dopamine following NO synthesis or an indirect pathway following the oxidation of the nitrite anion (NO_2_^-^) generated by the decay of NO to the nitrogen dioxide radical (NO_2_^-^), as demonstrated with mammalian heme peroxidases (16) and myeloperoxidase (17), remains to be established. Indeed, hydrogen peroxide (H_2_O_2_) is produced by the endothelium and causes vasodilatation (18) being possible that H_2_O_2_ enhances dopamine nitrosation/nitration. The finding that 6-ND release was strongly reduced by mechanical removal of the endothelium, as observed in other vascular tissues (4), further confirmed the endothelium as the major source for this catecholamine. Indeed, neither the marmoset’s thoracic aorta nor pulmonary artery present nerve terminals, as demonstrated by the absence of immunoreactivity for the neuronal markers S-100 (19,20) and calretinin (21,22).

In pre-contracted human umbilical vessels (4) and in *Chelonoidis carbonarius* aortic rings (5), the relaxations induced by 6-ND were similar to those induced by the dopamine D_2_-like receptor antagonist haloperidol, since they were not affected by pre-incubation with L-NAME but strongly reduced in endothelium-denuded rings. Dopamine is released by cultured endothelial cells (23), human umbilical cord arteries and vein (24), and *Chelonoidis carbonaria* aortic rings (25). Indeed, immunohistochemistry for tyrosine hydroxylase of both *Crotalus durissus terrificus* and *Bothrops jararaca* aortae revealed that this enzyme is present in endothelial cells (26). Immunohistochemistry for tyrosine hydroxylase was also positive in endothelial cells of *Chelonoidis carbonaria* aorta (27), and the presence of tyrosine hydroxylase and dopa-decarboxylase in the endothelial cells was further demonstrated in human umbilical artery and human umbilical vein, using both immunohistochemistry and fluorescence *in situ* hybridization (24). The results obtained with the L-741,626 indicated that the dopamine D_2_ receptor must play a major role in the control of vascular reactivity. Although the potency of 6-ND in the pre-contracted thoracic aorta rings (pEC_50_ 7.64±0.15) is nearly identical to that observed with the selective D_2_ receptor antagonist L-741,626 (pEC_50_ 7.70±0.15) (28), 6-ND has a remarkable selectivity for the dopamine receptors, since it did not affect the concentration-response curves to noradrenaline and adrenaline. Although 6-ND has been described as a reversible, competitive inhibitor of neuronal nitric oxide synthase (nNOS) (29), this mechanism is unlikely to be relevant for the 6-ND vasorelaxant action, since the inhibition of nNOS was seen at 45 μM whereas the EC_50_ for 6-ND-induced relaxations were 7.78±0.06 and 8.1±0.12 (that correspond to 63 and 12 nM, respectively) for pulmonary artery and aorta, respectively. Furthermore, one would expect inhibition of nNOS to cause vasoconstriction rather than vasorelaxation.

The dopamine D_2_-like antagonists do interact with adrenergic receptors, and the differences in potency (k_i_) for haloperidol (1.4 and 4.7 nM, for D_2_ and α_1_-adrenoceptor, respectively) and risperidone (2.2 and 1.4 nM, for D_2_ and α_1_-adrenoceptor, respectively) are discrete (30,31). Replacement of the NO_2_ group in the aromatic ring by other compounds such as Br, Cl, or CN may provide useful information on the development of “truly” selective dopamine D_2_-receptor antagonists. It is possible that this remarkable selectivity is restricted to 6-ND, since racemic 6-nitronoradrenaline acts as a weak α_1_-adrenoceptor agonist in the rat aorta (32). Our results indicated that 6-ND should be regarded as the first “truly” selective dopamine antagonist.

The finding that 6-ND inhibited EFS-induced contractions reinforced the novel concept of endothelium-derived catecholamines as main modulators of vascular reactivity (4,5,33). EFS causes contractions in vessels that are devoid of nerve terminals, such as human umbilical cord vessels (24), *Chelonoidis carbonaria* aortic rings (25,27), and *Panterophis guttatus* aortic rings (6). Since the contractions were strongly reduced by removal of the endothelium, it should be considered that endothelial cells in the vessel are excitable. The mechanism responsible for the contraction is the release of endothelium-derived dopamine and the attenuation of the EFS-induced contractions caused by 6-ND is due to the antagonism of dopamine at the D_2_- and/or D_2_-like receptors. Five genes encoding dopamine receptors have been identified, and the receptors are classified in two sub-families, namely the D_1_-like receptor subtypes (D1R and D5R), coupled to Gs, activating adenylyl cyclase and the D_2_-like subfamily (D2R, D3R, and D4R), coupled to Gi, inhibiting adenylyl cyclase (34). All five dopamine receptors have been identified in vascular beds *in vitro* by radioligand binding, autoradiographic techniques, and immunohistochemical analysis (35). Although dopamine is exogenously administered to maintain blood pressure and heart rate, its action is supposed to be on both α and β-adrenoceptors (36,37). Even the vasodilatory activity of the D_1_-like-receptor agonist fenoldopam (38) is supposed to be due to α_1_-adrenoceptor blockade in the kidney (39). The discovery that both dopamine and 6-ND are continuously released from vascular tissues and the finding that 6-ND acts as a “truly” selective dopamine antagonist should provide a fair reassessment of the role of this balance in the modulation of vascular reactivity.

Another novel finding was the potentiation of the EFS-induced contractions by L-NAME, vis-è-vis the lack of effect of the heme-site inhibitor of the soluble guanylate cyclase ODQ (40). The main mechanism proposed for the vasorelaxation induced by NO is stimulation of soluble guanylate cyclase (7), but this novel finding clearly reinforces the concept that the main mechanism for the vasorelaxation induced by NO is the synthesis of 6-ND (4,5). Indeed, this concept is further supported by the lack of effect of ODQ *in vivo*; administration of ODQ to rats did not affected MABP or heart rate, although *ex-vivo* inhibition of soluble guanylate cyclase was confirmed (40).

### Conclusion

Endothelium-derived 6-ND is the major catecholamine released from thoracic aorta and pulmonary artery rings and constitutes the major mechanism by which NO causes vasodilation.
